# Timing for Minimally Invasive Surgery in Lipoleiomyoma: Insights From Two Cases

**DOI:** 10.1155/crog/3667765

**Published:** 2026-02-10

**Authors:** Yoko Suzuki, Sakura Kataoka, Misato Ueda, Natsuki Nagashima, Asuka Yoshiara, Hidetaka Sato, Naoko Nakazawa

**Affiliations:** ^1^ Department of Obstetrics and Gynecology, Tokyo Metropolitan Police Hospital, Tokyo, Japan, keisatsubyoin.or.jp; ^2^ Departmemt of Obstetrics and Gynecology, Faculty of Medicine, The University of Tokyo, Tokyo, Japan, u-tokyo.ac.jp

**Keywords:** hysterectomy, laparoscopic surgery, leiomyoma, lipoleiomyoma, minimally invasive surgery, myomectomy

## Abstract

Lipoleiomyoma, a subtype of leiomyoma, may demonstrate hypertrophic growth, leading to tumor enlargement and presenting with clinical features such as postmenopausal bleeding and symptoms mimicking malignancy. These characteristics highlight the importance of careful evaluation and management in outpatient settings to ensure appropriate treatment. We report two cases of lipoleiomyoma with classical scenarios. The first case was initially misdiagnosed as an ovarian tumor and managed conservatively. Following tumor enlargement, laparoscopic surgery was performed after shared decision‐making with the patient. The second case mimicked malignancy and was managed surgically via laparotomy. These cases highlight typical clinical scenarios of lipoleiomyoma. From our first case to April 2024, a review of 50 reported cases revealed that only 12 were managed using minimally invasive surgery (MIS) despite the benign nature of the condition and the widespread accessibility of this surgical approach. Upon diagnosis, providing patients with these characteristics and possible disease course and management strategies is crucial. A less invasive laparoscopic approach is safe and feasible when appropriate preoperative evaluation and meticulous surgical planning are conducted.

## 1. Introduction

Uterine lipoleiomyoma is a benign subtype of leiomyoma. The incidence rate has been reported to be about 0.03%–0.28% [1]. Once a lipoleiomyoma is diagnosed, it can be treated in the same way as a uterine leiomyoma. Asymptomatic cases typically do not require interventions. Although minimally invasive surgery (MIS) is generally preferred, a substantial proportion of reported cases ultimately require laparotomy because tumors enlarge during follow‐up or cannot be clearly distinguished from malignancy. We present two cases that highlight how these common challenges affect the timing and feasibility of MIS.

## 2. Case Presentation

### 2.1. Case 1

A 55‐year‐old postmenopausal nulliparous woman underwent a medical checkup 4 months ago, revealing a left ovarian mass. She was asymptomatic during her visit to our department. Her medical history includes hypertension and glaucoma.

#### 2.1.1. Imaging Findings

Transvaginal ultrasonography showed a 4 × 3 × 2 cm hyperechoic (Figure 1a), well‐circumscribed mass near the left uterus with no vascularity (4.1), initially thought to be an ovarian tumor with a fatty component. MRI later identified it as a subserosal uterine lipoleiomyoma leading to a conservative follow‐up (Figure 1c).

Figure 1(a) Transvaginal ultrasound of first visit; (b) transvaginal ultrasound 1 year after during follow‐up; (c) MRI; (d) gross pathology; and (e) tissue pathology (H&E, X400).

**Figure:**
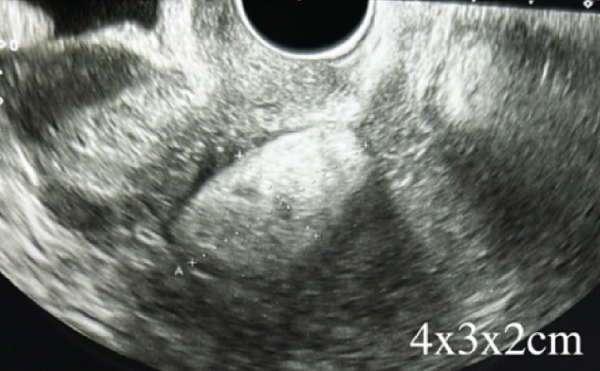
(a)

**Figure:**
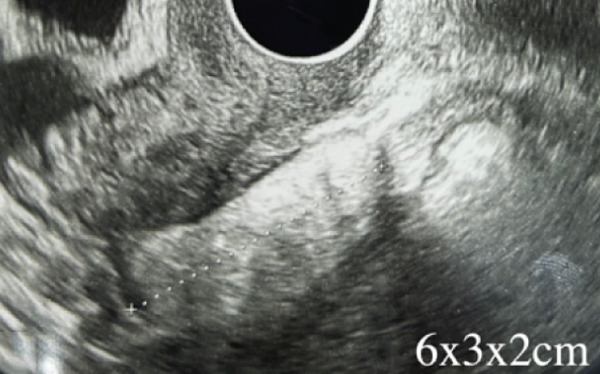
(b)

**Figure:**
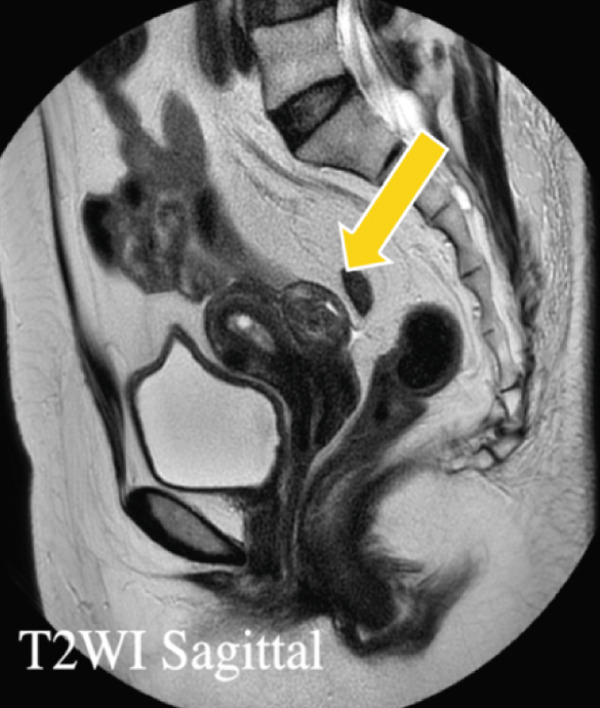
(c)

**Figure:**
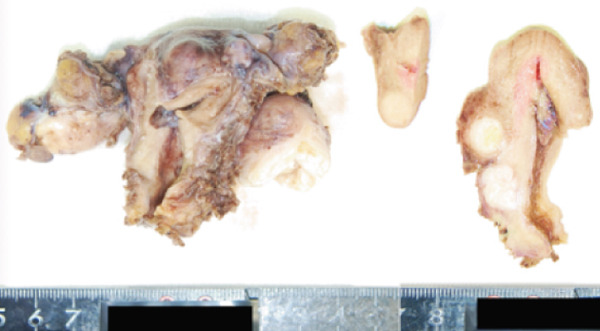
(d)

**Figure:**
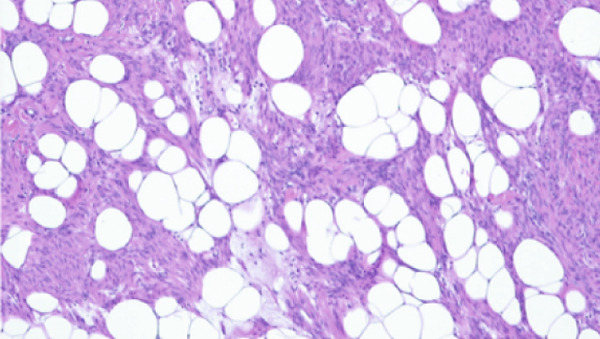
(e)

Over a year, the mass increased to 6 × 3 × 2 cm (Figure 1b). After reviewing the diagnosis and discussing options, the patient requested surgery.

#### 2.1.2. Examination Findings

Preoperative exams revealed Type 2 diabetes mellitus and hyperlipidemia, leading to a referral to internal medicine for medication. The ophthalmologist cautioned that surgery might elevate intraocular pressure, exacerbating glaucoma due to the Trendelenburg position. Robotic hysterectomy typically requires a 25°–40° head‐down tilt, whereas laparoscopic surgery often maintains less than 20°.

#### 2.1.3. Management

To manage her glaucoma risk, a laparoscopic total hysterectomy with bilateral salpingo‐oophorectomy was chosen. Pathology: postoperative pathology confirmed a well‐circumscribed fatty mass with multiple tan‐white whorled cut surfaces in the myometrium (Figure 1d) and scattered lipid droplets in the leiomyoma (Figure 1e). The patient recovered smoothly without complications, including no changes in glaucoma status.

### 2.2. Case 2

A 49‐year‐old nulliparous woman presented at the hospital with a complaint of abnormal genital tract bleeding lasting for 18 days, accompanied by a notable escalation in bleeding over the preceding 10 days. Pelvic examination revealed an intra‐abdominal mass up to the umbilicus.

#### 2.2.1. Imaging Findings

Transvaginal ultrasound showed a heterogeneous echo of more than 10 cm mass in the anterior wall of the uterus.

Contrast‐enhanced CT imaging revealed a dense mass measuring 66 × 104 mm in the anterior wall of the uterus and a tumor located in the upper pole of the right kidney, exhibiting a heterogeneous enhancement (Figure 2a).

Figure 2(a) Contra‐enhanced CT and (b) gross pathology.

**Figure:**
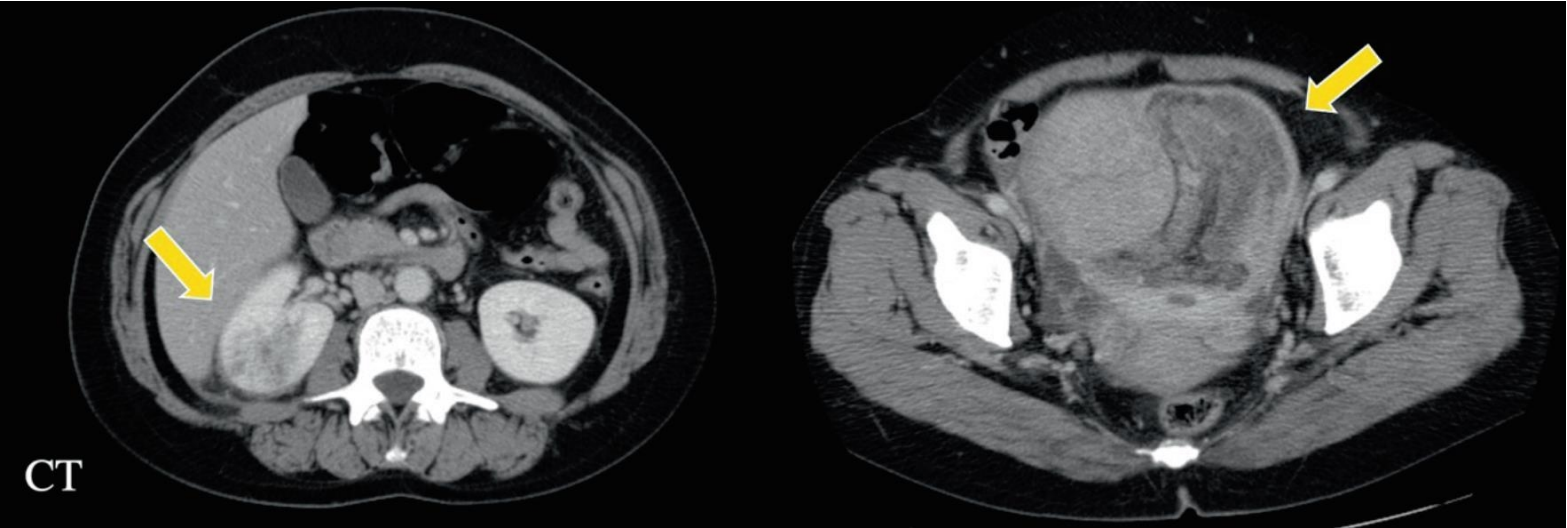
(a)

**Figure:**
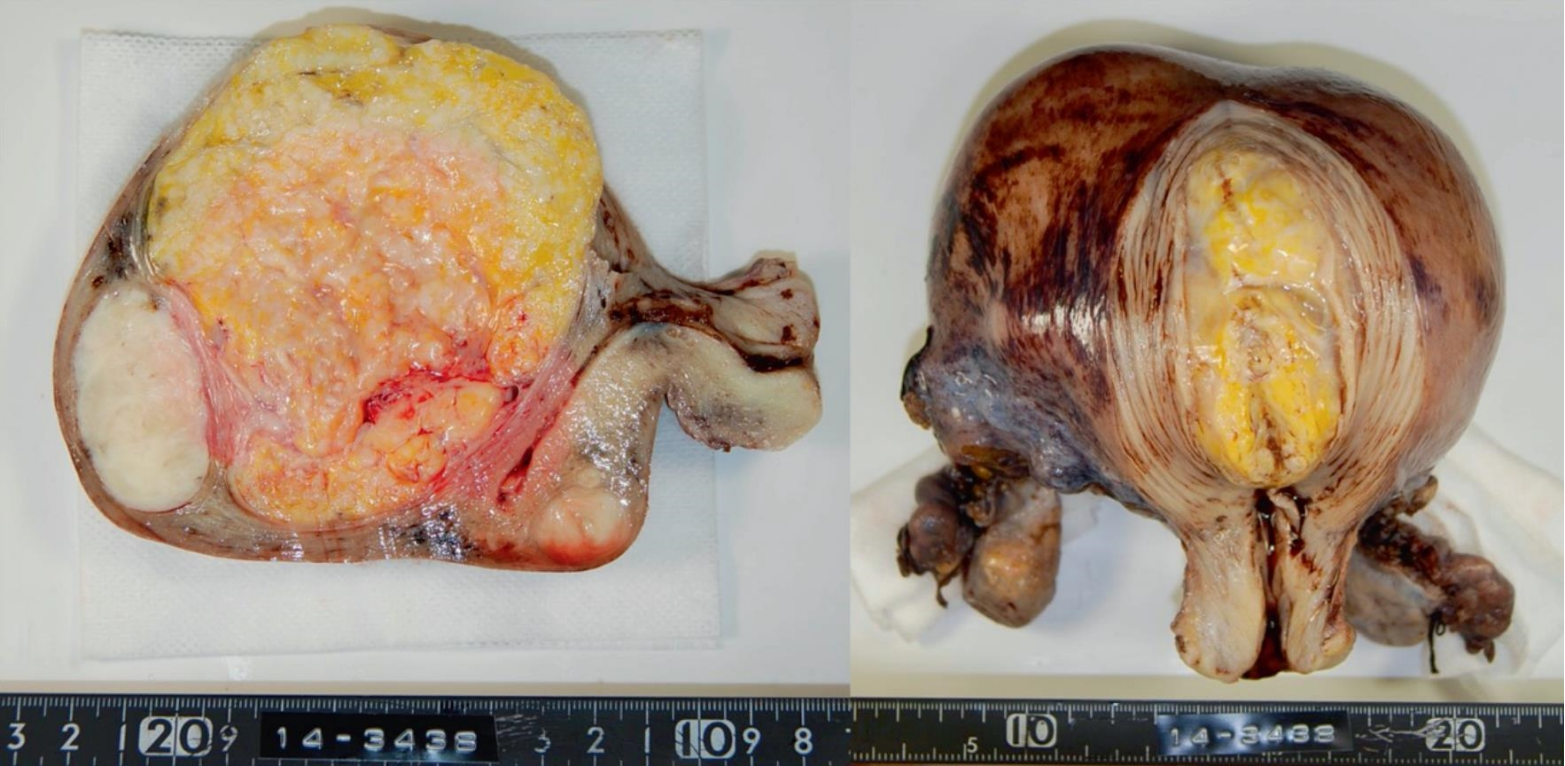
(b)

A dynamic contrast‐enhanced CT of the kidney was conducted 3 days after admission. A 26 mm hypovascular invasive tumor was once more observed in the right kidney, suggesting differential diagnoses of duplicate malignancies such as malignant lymphoma, Bellini′s tubular carcinoma, or a metastatic lesion from the uterine tumor.

MRI could not be conducted due to the patient′s claustrophobia.

#### 2.2.2. Examination Findings

Cervical cytology and endometrial histology were obtained. The patient presented with a temperature of 39°C, a WBC count of 11,400/*μ*L, and a CRP level of 13.9 mg/dL. Blood culture revealed gram‐negative bacilli, diagnosing bacteremia.

#### 2.2.3. Management

Based on clinical findings, we first suspected a sarcoma. As inflammation subsided, a total hysterectomy with bilateral salpingo‐oophorectomy was performed via a median longitudinal incision on the 10th day postadmission. The 1‐hour, 41‐minute operation had a 150 ml blood loss, and the specimen weighed 1050 g. She recovered uneventfully and was discharged on the 8th day.

#### 2.2.4. Pathology

Postoperative pathology showed a 10 cm yellow, soft tumor and coexisting uterine leiomyomas (Figure 2b). Histology confirmed lipoleiomyoma with mature adipose tissue.

Shortly after discharge, a comprehensive kidney tumor evaluation, including urine cytology and contrast‐enhanced CT, showed reduced hypoenhanced areas consistent with inflammatory pyelonephritis.

## 3. Discussion

### 3.1. Presented Two Case

Lipoleiomyoma predominantly affects postmenopausal women [2]. The edematous or hypertrophic changes can lead to tumor growth that mimics malignancy—often misdiagnosed with an ovarian tumor before surgery if MRI is not implemented. In addition, these tumors grow more rapidly than conventional uterine leiomyomas, complicating treatment decision‐making compared with usual leiomyomas [3].

In our institution, only two cases mentioned above were pathologically confirmed lipoleiomyoma among 798 cases of uterine leiomyoma in the past 15 years (0.25%, Table 1). These cases illustrate key diagnostic and management challenges.

**Table 1:** Case 1 and Case 2 information.

Feature	Case 1	Case 2
Age	55‐year‐old	49‐year‐old
Parity	Nulliparous	Nulliparous
Menopause	Postmenopausal	Premenopausal
Chief Complaint	Incidental finding during medical checkup	Abnormal genital bleeding for 18 days, fever(39 C)
Initial diagnosis	Left ovarian tumor with fatty components	Uterine sarcoma and coexisting kidney tumor
Ultrasound	Hyperechoic well‐circumscribed mass	Heterogeneous uterine mass
Imaging	MRI: 4 × 3 × 2 cm subserous uterine lipoleiomyoma	CT: 10.4 × 6.6 cm uterine mass and 2.6 cm kidney tumor
Complication	Hypertension, glaucoma, Type 2 diabetes mellitus, hyperlipidemia	Bacteremia (gram‐negative bacilli), suspected pyelonephritis
Surgery	TLH + BSO	TAH + BSO (median longitudinal incision)
Pathology	Lipoleiomyoma	Lipoleiomyoma, coexisting uterine leiomyomas

Abbreviations: Abd, Abdominal; AUB, Abnormal uterine bleeding; BSO, bilateral salpingo‐oophorectomy; TAH, transabdominal hysterectomy; TLH, translaparoscopic hysterectomy.

The first case, initially misdiagnosed as an ovarian tumor, was correctly identified as a uterine leiomyoma via MRI. If diagnosed earlier as a leiomyoma, follow‐up might have been deemed unnecessary, potentially missing the timing for MIS.

The second case, a preexisting uterine leiomyoma, unmonitored due to menopause, coincided with an infection. Claustrophobia prevented an MRI, leading to insufficient information to rule out malignancy, resulting in a longitudinal incision that later revealed benign pathology.

These cases demonstrate some classical scenarios, as reported in a case series study in which 16% of all cases were diagnosed with another disease, such as teratoma [4]. Differential diagnosis from malignancy is occasionally problematic, especially when contrast‐enhanced MRI is unavailable, and it hinders the application of MIS [5].

## 4. Literature Review

To contextualize our experience, we reviewed the literature on lipoleiomyomas, including reports that specifically mentioned treatment, in PubMed, MEDLINE, and Web of Science, and identified 50 studies. Among these reports, despite this condition′s benign nature and MIS′s widespread availability, only 12 cases were reported as having been managed using MIS [6–14]. Seven laparoscopic hysterectomies, four laparoscopic tumor resections, and one robotic‐assisted hysterectomy are reported (Table 2). This reflects the situation in which most cases missed the appropriate timing for MIS despite the benign nature of the condition and the widespread availability of this surgical approach.

**Table 2:** Demographics of case reports.

Year	Author	Age	Symptoms	Parity	Size	MP	Surgery
2010	[6] Ding DC et al.	60	Abnormal vaginal bleeding for 3 months	NA	5.6 × 4.4	51	Dilatation and curettage + removal of the device + TLH
2015	[7] Oh SR et al.	55	Hot flashes	3	4 × 3.5, 2.4 × 1	No	TLH + BSO
		45	Abd discomfort	1	21 × 18	No	TLH + BSO
2022	[8] Kim YS et al.	55	4 cm size 4 years ago, increase to 5.8 cm in the last year	2	6.8 × 4.5 × 0.6	50	Laparoscopic resection + BSO
2022	[9] Wang YY et.al	38	Abd Pain, AUB, pregnancy	3	7 × 6	No	Laparoscopic resection after 45 days of abortion
2022	[10] Wan YZ et.al	58	Increased urinary frequency	NA	10 × 7 × 8	Yes	TLH + BSO
		58	AUB	NA	1.5 × 1.4 × 1.7	Yes	TLH + BSO
		45	Hypermenorrhea	NA	5.7 × 5.2 × 3.4	No	TLH + BS
2023	[11] Bandarian M et al.	55	Vaginal bleeding	NA	8.0 × 6.0	Yes	TLH
2023	[12] Limaiem F et al.	57	6‐month history of pelvic pain	5	4 × 3 × 2.5 cm	Yes	Laparoscopic resection
2023	[13] Chen G et al.	47	Health checkup, asymptomatic	NA	3.8 cm × 3.5 cm × 3.0 cm	No	Laparoscopic resection
2023	[14] Tam T et al.	53	AUB and difficulty emptying her bladder	0	8.8	No	RALH

Abbreviations: BS, bilateral salpingectomy; BSO, bilateral salpingo‐oophorectomy; RALH, robotic assisted laparoscopic hysterectomy; TAH, transabdominal hysterectomy; TLH, translaparoscopic hysterectomy.

Bosoteanu et al. discuss a case of a giant uterine tumor identified as a lipoleiomyoma, emphasizing their potential to reach a significant size if left untreated [15]. The case for adopting laparoscopic approaches, even for larger uterine sizes previously considered unsuitable, has been strengthened by technical advancements and growing surgical expertise; the size of the uterus is no longer an absolute contraindication to endoscopic surgery.

Yuan et al. [5] provide an exhaustive literature review on the pathogenesis, diagnosis, and treatment of uterine lipoleiomyomas, detailing the role of imaging and biopsy in accurate diagnosis and the consideration for surgery in symptomatic cases or when the diagnosis is uncertain. In Japan, preoperative MRI is performed in almost all cases. Contrast‐enhanced MRI is performed when differentiation from malignancy is required based on clinical symptoms or transvaginal ultrasound findings. On the other hand, a uterine leiomyoma, without indication for surgery, would not have a regular MRI. Hence, if a leiomyoma with a significant adipose component is detected on transvaginal ultrasound, it is preferable to obtain an MRI to confirm the diagnosis. Although some reports indicate that MRI may fail to diagnose specific types with dispersed or low concentrations of fatty components [4], the tool remains a powerful tool in identifying adipose tissues.

**Figure 3:**
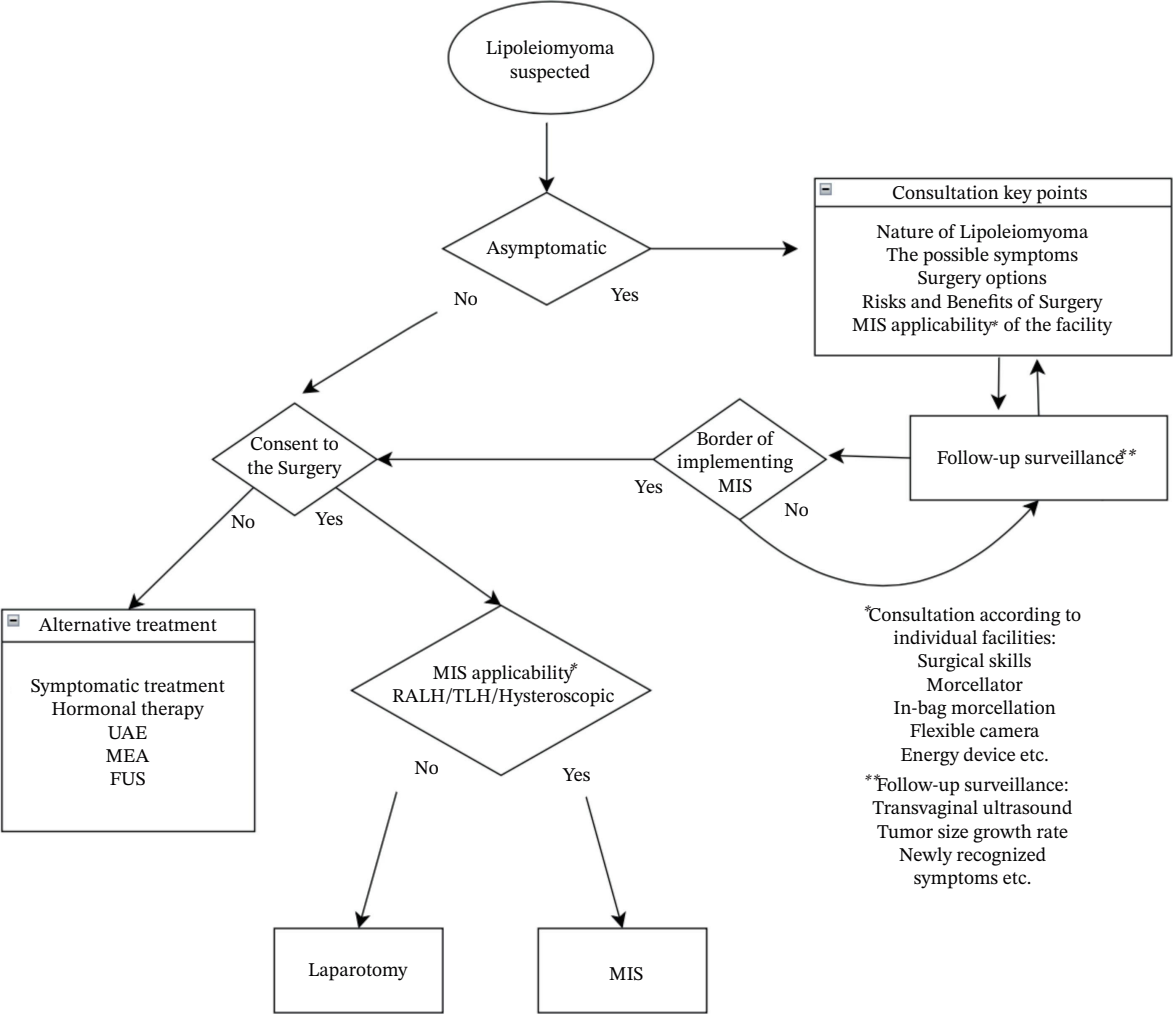
Proposed flow chart of shared decision‐making in lipoleiomyoma patients and physicians.

Needless to say, MIS offers many advantages over traditional laparotomy. However, the choice between MIS and laparotomy often depends on the medical center′s policy. When retrospectively reviewing cases, some patients were initially diagnosed at a smaller stage, making them suitable candidates for MIS. Therefore, it is essential to inform patients about the institution′s capability and any size limitations associated with MIS when diagnosing lipoleiomyoma. Providing thorough informed consent facilitates shared decision‐making between physicians and patients. Ensuring patients are actively involved in choosing the most appropriate treatment approach is essential. The clinical workflow illustrated in Figure 3 underscores the versatility of laparoscopic techniques, enabling optimal tumor resection while minimizing patient burden.

The following situations require special attention. In small asymptomatic cases, follow‐up is often discontinued after menopause; however, when ultrasound suggests a substantial fatty component, clinicians should make an effort to obtain an MRI and provide appropriate management guidance. Whereas in large symptomatic lipoleiomyomas—such as cases requiring urgent intervention for severe hemorrhage—the presence of significant adipose tissue should prompt clinicians to consider lipoleiomyoma in the differential diagnosis whenever possible and to avoid excessive or overly aggressive treatment when this benign condition is likely.

Finally, recent advances in AI‐based image recognition may further improve the accuracy of preoperative diagnosis for lipoleiomyoma.

## 5. Limitation

Case reports often focus on unique cases or those involving specific treatments, underrepresenting cases that underwent conservative management or follow‐up; it remains unknown how many of the cases under follow‐up would eventually necessitate surgery.

## 6. Conclusion

Uterine lipoleiomyoma is a benign leiomyoma subtype that may exhibit hypertrophic growth, causing tumor enlargement, postmenopausal bleeding, and malignancy‐like symptoms. MRI is vital for accurate diagnosis. Treatment is generally unnecessary for asymptomatic cases, but surgical decisions depend primarily on lesion size and the facility′s MIS capabilities. Timely surgical intervention is crucial to prevent progression that could complicate surgery or recovery.

## Funding

No funding was received for this manuscript.

## Consent

No written consent has been obtained from the patients as there is no patient identifiable data included in this case report.

## Conflicts of Interest

The authors declare no conflicts of interest.

## Data Availability

Data sharing is not applicable to this article as no new data were created or analyzed in this study.
